# Medical Application of Functional Biomaterials—The Future Is Now

**DOI:** 10.3390/jfb13040244

**Published:** 2022-11-16

**Authors:** Cristian Scheau, Andreea Cristiana Didilescu, Constantin Caruntu

**Affiliations:** 1 Department of Physiology, The “Carol Davila” University of Medicine and Pharmacy, 050474 Bucharest, Romania; 2 Department of Embryology, Faculty of Dentistry, The “Carol Davila” University of Medicine and Pharmacy, 050474 Bucharest, Romania; 3 Department of Dermatology, “Prof. N.C. Paulescu” National Institute of Diabetes, Nutrition and Metabolic Diseases, 011233 Bucharest, Romania

We live in unprecedented times. Technology and manufacturing capabilities worldwide are rapidly expanding as new devices, substances, and materials are developed to fulfill the needs of different areas of interest, including medicine. Functional biomaterials have flooded the medical field in multiple domains with numerous applications, from replacing tissues and organs to consolidating regional physiology by promoting bone mineralization, balancing regional flora, and facilitating regeneration [1,2,3].

Polydimethylsiloxane (PDMS) is a widely used polymer with excellent biocompatibility, stability, and mechanical properties. Miranda et al. have presented an excellent review of recent applications of PDMS, a walkthrough of the properties and manufacturing process of this polymer, as well as the various medical applications of this substance found in recent literature, such as its use as a blood analogue, in implant coatings, or in replicating the cardiovascular system [4]. The authors also considered the microfabrication process of PDMS, which was thoroughly described, and further provided the best methods to characterize this compound and test its properties according to manufacturing standards. Auliya et al. have tested PDMS for its potential use as a vitreous substitute in the human eye [5]. In this study, compounds with viscosities of 1.15 Pa.s, 1.17 Pa.s, and 1.81 Pa.s were created, and the authors evaluated the physical properties of the mentioned samples. Furthermore, in vitro tests were performed to measure the toxicity of this material using the Hen’s Egg Test Chorioallantoic Membrane method for the first time. Ultimately, it was shown that PDMS is safe and may replace the vitreous humor in human eyes—a very encouraging finding.

Titanium–nickel alloys have long been used in medical devices and implants, with papers going back to the 1980s citing the use of TiNi alloys, especially in the dental field [6]. However, recent advances in the manufacturing process have led to the development of porous TiNi alloys through self-propagating high-temperature synthesis, a material able to sustain the attachment and growth of human cells with great biocompatibility while maintaining excellent mechanical properties [7]. Topolnitskiy et al. have tested the performance of these types of implants in the repair of the chest wall after resection for malignant tumors [8]. The authors concluded that the material is safe and has good functional properties using one-stop surgery.

Stimulation of the body’s regenerative properties is essential in healing after implant placement, and using appropriate substances is essential in favoring the acceptance of the newly introduced biomaterials. Bleyan et al. have shown that osseodensification may be used as a predictor for immediate implant placement using allografts or alloplasts as fillers [9]. They also proposed a new classification of the molar socket that takes into consideration the septum width before instrumentation and can be applied to estimate the impact on implant stability; according to the authors, S-IV sockets have less than 2 mm of initial septum width and are counterindications for septum expansion with osseodensification. The study of Srisomboon et al. assessed the restorative properties of silver diamine nitrate (SDN) and fluoride (SDF) on dentin remineralization while accounting for their cytotoxicity to dental pulp [10]. The authors proposed SDN as a more cost-effective alternative and developed a study to measure its effectiveness compared to SDF. SDF was shown to be more efficient than SDN in increasing apatite formation while showing similar cytotoxic effects on dental pulp cells. However, both compounds showed comparable precipitation of silver salts in the demineralized dentin, occluding the dentinal tubules; therefore, SDN was proposed as a potential material for caries control.

On a similar topic, Covaci et al. tested various calcium-based materials in deep carious lesions to assess their potential in maintaining dental pulp vitality [11]. The study revealed that self-setting calcium hydroxide was superior in the preservation of dental pulp vitality compared to resin-modified, calcium-releasing compounds. In addition, the pH of the compound was shown to be a factor that can affect the dental pulp and trigger inflammation or necrosis. These results advance the knowledge base on the biomaterials used in direct pulp capping, as the ideal material is still being sought.

Controlling the local environment around implants, prostheses, or other medical devices is key to preventing adverse reactions such as inflammation, infection, or various immune responses. In their study, Chanachai et al. prepared an adhesive containing monocalcium phosphate monohydrate (MCPM) and nisin to be used in fixed orthodontic treatment [12]. The material is strong enough to prevent bracket debonding and releases calcium phosphate, encouraging mineralization and buffering the local acidity while showing antibacterial properties. After a thoughtful interpretation of their results, the authors have proposed updated models of their developed compound that might have increased effectiveness in all these regards.

Moreover, the importance of biocompatibility and molecular interaction is underlined by Tizu et al. in their paper, where they elaborate on the interaction between two different polymeric nanoparticles and oral cells, more specifically, stem cells of keratinocyte as well as human exfoliated deciduous teeth [13]. The authors demonstrate the baseline for the polymer’s uptake and note various significant parameters of the process. The findings are very relevant in terms of developing novel methods for tissue regeneration with reduced toxicity and high efficacy.

This Special Issue showed that the research field of functional biomaterials is of tremendous interest and importance, with rapid evolution and numerous applications. For medical usage of new biomaterials, it is required that safety and biocompatibility standards are met alongside the demonstration of the superior physical and chemical properties of the compounds. Adequate integration of biomaterials in complex biological environments that are thoroughly regulated by a wide array of physiological mechanisms will always represent a challenge for future developers of compounds, alloys, and substances. In the very first editorial of this Journal, the Founding Editor-in-Chief recognized the incredible potential of the area of biomaterials, the increasing scientific interest, as well as the multidisciplinarity involved in the development of this field [14]. It is our hope that our Special Issue will help advance the current knowledge of the medical applications of functional biomaterials and that it represents a proper forum for the intersection and cooperation of the scientific and technological communities, as Prof. Francesco Puoci intended for this Journal.

## Data Availability

Not applicable.

## References

[1] Riedel S., Ward D., Kudláčková R., Mazur K., Bačáková L., Kerns J.G., Allinson S.L., Ashton L., Koniezcny R., Mayr S.G. (2021). Electron Beam-Treated Enzymatically Mineralized Gelatin Hydrogels for Bone Tissue Engineering. J. Funct. Biomater..

[2] Iovene A., Zhao Y., Wang S., Amoako K. (2021). Bioactive Polymeric Materials for the Advancement of Regenerative Medicine. J. Funct. Biomater..

[3] Hamdan N., Yamin A., Hamid S.A., Khodir W., Guarino V. (2021). Functionalized Antimicrobial Nanofibers: Design Criteria and Recent Advances. J. Funct. Biomater..

[4] Miranda I., Souza A., Sousa P., Ribeiro J., Castanheira E.M.S., Lima R., Minas G. (2022). Properties and Applications of PDMS for Biomedical Engineering: A Review. J. Funct. Biomater..

[5] Auliya D.G., Setiadji S., Fitrilawati F., Risdiana R. (2022). Physical Characterization and In Vitro Toxicity Test of PDMS Synthesized from Low-Grade D4 Monomer as a Vitreous Substitute in the Human Eyes. J. Funct. Biomater..

[6] Kimura H., Sohmura T. (1987). Surface coating on TiNi shape memory implant alloys. J. Osaka Univ. Dent. Sch..

[7] Yasenchuk Y., Marchenko E., Baigonakova G., Gunther S., Kokorev O., Gunter V., Chekalkin T., Topolnitskiy E., Obrosov A., Kang J.-H. (2021). Study on tensile, bending, fatigue, and in vivo behavior of porous SHS–TiNi alloy used as a bone substitute. Biomed. Mater..

[8] Topolnitskiy E., Chekalkin T., Marchenko E., Yasenchuk Y., Kang S.-B., Kang J.-H., Obrosov A. (2021). Evaluation of Clinical Performance of TiNi-Based Implants Used in Chest Wall Repair after Resection for Malignant Tumors. J. Funct. Biomater..

[9] Bleyan S., Gaspar J., Huwais S., Schwimer C., Mazor Z., Mendes J.J., Neiva R. (2021). Molar Septum Expansion with Osseodensification for Immediate Implant Placement, Retrospective Multicenter Study with Up-to-5-Year Follow-Up, Introducing a New Molar Socket Classification. J. Funct. Biomater..

[10] Srisomboon S., Kettratad M., Stray A., Pakawanit P., Rojviriya C., Patntirapong S., Panpisut P. (2022). Effects of Silver Diamine Nitrate and Silver Diamine Fluoride on Dentin Remineralization and Cytotoxicity to Dental Pulp Cells: An In Vitro Study. J. Funct. Biomater..

[11] Covaci A., Ciocan L.T., Gălbinașu B., Bucur M.V., Matei M., Didilescu A.C. (2022). Dental Pulp Response to Different Types of Calcium-Based Materials Applied in Deep Carious Lesion Treatment&mdash; a Clinical Study. J. Funct. Biomater..

[12] Chanachai S., Chaichana W., Insee K., Benjakul S., Aupaphong V., Panpisut P. (2021). Physical/Mechanical and Antibacterial Properties of Orthodontic Adhesives Containing Calcium Phosphate and Nisin. J. Funct. Biomater..

[13] Tizu M., Mărunțelu I., Cristea B.M., Nistor C., Ishkitiev N., Mihaylova Z., Tsikandelova R., Miteva M., Caruntu A., Sabliov C. (2022). PLGA Nanoparticles Uptake in Stem Cells from Human Exfoliated Deciduous Teeth and Oral Keratinocyte Stem Cells. J. Funct. Biomater..

[14] Puoci F. (2010). Salve Journal of Functional Biomaterials, ad maiòra!. J. Funct. Biomater..

